# Impact of carboplatin schedule on pCR in a modified KEYNOTE-522 regimen with dose-dense AC for triple negative breast cancer

**DOI:** 10.1093/oncolo/oyaf372

**Published:** 2025-11-19

**Authors:** Kennedi G Satterfield, Michael Berger, Allison Reed, Eric McLaughlin, Stephanie Collins, Dionisia Quiroga

**Affiliations:** The Arthur G. James Cancer Hospital and Richard J. Solove Institute at The Ohio State University, Department of Pharmacy, Columbus, OH 43210, United States; The Arthur G. James Cancer Hospital and Richard J. Solove Institute at The Ohio State University, Department of Pharmacy, Columbus, OH 43210, United States; The Arthur G. James Cancer Hospital and Richard J. Solove Institute at The Ohio State University, Department of Pharmacy, Columbus, OH 43210, United States; The Ohio State University College of Medicine, Center for Biostatistics, Columbus, OH 43210, United States; The Arthur G. James Cancer Hospital and Richard J. Solove Institute at The Ohio State University, Department of Pharmacy, Columbus, OH 43210, United States; The Ohio State University Wexner Medical Center, Division of Medical Oncology, The Stefanie Spielman Comprehensive Breast Center, Columbus, OH 43212, United States

**Keywords:** Triple Negative Breast Neoplasms, Neoadjuvant Therapy, Pathologic Complete Response, Carboplatin, Paclitaxel, Pembrolizumab

## Abstract

**Background:**

As triple-negative breast cancer (TNBC) has a poorer prognosis when compared to hormone- and human epidermal growth factor 2-positive disease, it is vital to find treatments and schedules to improve outcomes inpatients with TNBC. The purpose of this study is to compare pathologic complete response (pCR) rates in early-stage TNBC between weekly versus every three-week carboplatin dosing with paclitaxel and pembrolizumab followed by dose-dense doxorubicin and cyclophosphamide (ddAC) and pembrolizumab in a modified KEYNOTE-522 regimen.

**Methods:**

A retrospective, single-center review was conducted on patients who received both treatment and surgery at the James Cancer Hospital at The Ohio State University Medical Center (The James) for TNBC with carboplatin, paclitaxel, and pembrolizumab followed by ddAC and pembrolizumab.

**Results:**

A total of 92 patients were included in this study; 51 patients received weekly carboplatin and 41 patients received every three-week carboplatin. The pCR rate at time of surgery was 47.1% in the weekly group and 70.1% (*P* = 0.03) for the every three-week group. Dose reductions of chemotherapy (35.3% vs. 26.8%) and dose delays of greater than 7 days due to immunotherapy toxicities (23.5% vs. 14.6%) were greater in the weekly cohort. There were no differences in grade 3 or higher neutropenia between groups nor the use of granulocyte colony stimulating factor support. Infusion hypersensitivity reactions (iHSR) occurred in 19.6% of weekly patients and 4.9% of every three-week patients (*P* = 0.06).

**Conclusion:**

In this single-center analysis, every three-week carboplatin dosing followed by ddAC in a modified KEYNOTE-522 regimen provides higher pCR rates at the time of surgery. Patients who received weekly carboplatin dosing experienced more iHSR. These findings strongly warrant additional studies to determine the relationship of carboplatin dosing to TNBC outcomes.

Implications for Practice The standard of care treatment for clinical stage II-III TNBC is neoadjuvant chemotherapy consisting of carboplatin, paclitaxel, and pembrolizumab, followed by doxorubicin and cyclophosphamide (AC) and pembrolizumab. KEYNOTE-522 allowed carboplatin administration either weekly or every three-week, followed by every three-week AC. Our study provides compelling data that when given with ddAC, providers should consider every three-week carboplatin with ddAC over a weekly carboplatin regimen for improved pCR rates and comparable adverse effect profiles, including the concern for myelosuppression. The risk of developing an iHSR is also lowered with every three-week carboplatin.

## Introduction

Breast cancer is the second leading cause of cancer-related deaths in women in the United States.^1^ Triple-negative breast cancer (TNBC) has a higher rate of recurrence and mortality when compared to hormone receptor (HR)-positive and human epidermal growth factor 2-positive breast cancers. Neoadjuvant (pre-operative) chemotherapy is used for the treatment of early-stage TNBC to decrease and eradicate disease in the breast and/or local lymph nodes prior to surgery with the goal of obtaining a pathologic complete response (pCR). Pathologic complete response is defined as pathologic stage “ypT0/Tis ypN0”, meaning there was no residual invasive disease detected in the breast or axilla at the time of definitive surgery. Pathologic complete response at the time of surgery following neoadjuvant chemotherapy is a favorable indicator of disease-free and overall survival, particularly for TNBC.^2^ Following an Alliance trial for stages II and III TNBC which demonstrated that the addition of carboplatin to neoadjuvant paclitaxel can significantly increase pCR rates in both the breast and axilla, the standard neoadjuvant chemotherapy for early-stage TNBC consisted of paclitaxel and carboplatin followed by doxorubicin and cyclophosphamide (AC).^3^

In 2020, the KEYNOTE-522 trial demonstrated that the addition of pembrolizumab to carboplatin and paclitaxel followed by AC improved pCR rates in patients with early clinical stage II to III TNBC.^4^ KEYNOTE-522 allowed treating physicians to choose if a patient received weekly or every three-week dosing of carboplatin along with weekly paclitaxel. Although KEYNOTE-522 used an every three-week schedule for AC, given the improved outcomes seen with ddAC in previous studies, some institutions have substituted ddAC for every three-week AC, thus creating a “modified” KEYNOTE-522 regimen.^5^ Given the unknown impact of weekly versus every three-week dosing of carboplatin on pCR rates in patients with TNBC receiving the modified KEYNOTE-522 regimen, we sought to retrospectively examine pCR rates in our patient population.

## Methods

### Study design and patient population

This was a single-center, retrospective, cohort study of patients who received both treatment and surgery at The Ohio State University Comprehensive Cancer Center—James Cancer Hospital & Solove Research Institute for TNBC with pembrolizumab, paclitaxel, and carboplatin followed by pembrolizumab and ddAC between July 1, 2021 and April 30, 2024. Patients were greater than 18 years of age and had clinical stage II or III TNBC or HR low-positive disease (defined as estrogen receptor [ER] and/or progesterone receptor [PR] 1%-10%) as defined by the guidelines of the American Society of Clinical Oncology–College of American Pathologists.^6^ Patients in both groups were routinely evaluated every three weeks on day 1 by the clinical team with blood draws that included a comprehensive metabolic panel and a complete blood count (CBC). Patients would then have a weekly assessment visit and a CBC collected on days 8 and 15 of each cycle. As such, the frequency and nature of assessments were similar across treatment groups, unless the care team deemed it was clinically necessary to increase visits and/or laboratory monitoring. During treatment with carboplatin and paclitaxel, patients were not required to receive primary prophylaxis with granulocyte colony stimulating factor (GCSF), as this is not standard practice at our institution, but it can be added if a neutropenic event is anticipated or occurs. Patients were identified for inclusion via electronic medical record report identifying the KEYNOTE-522 chemotherapy regimen during the study time frame. Patients were excluded if they were pregnant, incarcerated, had stage I or IV TNBC, or had ER or PR receptor positivity greater than 10%. Patients who discontinued chemotherapy prior to starting ddAC, did not receive ddAC, or had a planned scheduled change from ddAC to every three-week AC mid-treatment were also excluded. Study data were collected and managed using Research Electronic Data Capture electronic data capture tools hosted at The Ohio State University Wexner Medical Center.^7^^,^^8^ Research Electronic Data Capture is a secure, web-based software platform designed to support data capture for research studies, providing (1) an intuitive interface for validated data capture, (2) audit trails for tracking data manipulation and export procedures, (3) automated export procedures for seamless data downloads to common statistical packages, and (4) procedures for data integration and interoperability with external sources.

### Objectives

The primary objective of this study was to compare the pCR rate at the time of definitive surgery in patients who received weekly versus every three-week carboplatin dosing with paclitaxel and pembrolizumab followed by ddAC and pembrolizumab per the modified KEYNOTE-522 regimen. Pathologic complete response was evaluated by a pathologist based on the surgical pathology specimen. Patients who had residual non-invasive cancer, such as ductal carcinoma in-situ, were considered to have achieved a pCR. Secondary outcomes included residual cancer burden (RCB) score at the time of definitive surgery,^9^ any delays or dose reductions in chemotherapy or immunotherapy due to relative toxicity, total number of doses of chemotherapy or immunotherapy received, duration of treatments, occurrence of grade 3 or higher neutropenia, the use of GCSF during treatment with carboplatin and paclitaxel, and the occurrence of a carboplatin Infusion hypersensitivity reactions (iHSR), defined as an infusion hypersensitivity reaction necessitating the use of parenteral rescue medication(s) including hydrocortisone, diphenhydramine, famotidine or epinephrine.

### Statistical analysis

Demographics and clinical data were reported for the overall study cohort and compared between weekly and every-three-week dosing regimens using Fisher’s exact tests or Wilcoxon rank-sum tests. The primary outcome, pCR, was compared between treatment regimens by a Fisher’s exact test. The univariate analysis of pCR was also performed using Fisher’s exact tests for comparisons of categorical variables, and Wilcoxon rank-sum tests for comparisons of continuous variables, due to non-normal distributions of the data. Secondary outcomes were also compared between treatment regimens using these methods. Multivariable logistic regression was used to assess group differences in pCR rates while accounting for repeated measures with adjustment for confounding of age, grade, tumor size, and clinical stage. Statistical analyses were performed using SAS 9.4 (SAS Institute Inc., Cary, NC).

## Results

### Patient characteristics

**Figure 1.**
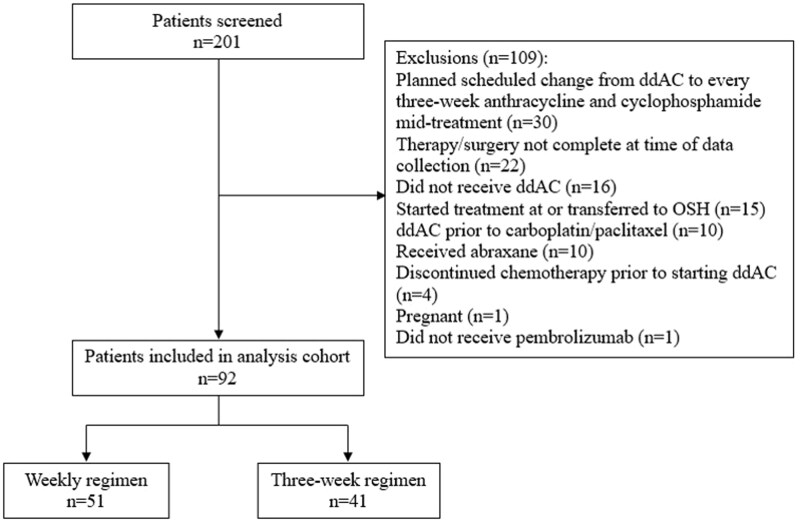
Consort diagram of patient exclusions. ddAC, dose-dense doxorubicin and cyclophosphamide; OSH, outside hospital.

From July 1, 2021 to April 30, 2024, a total of 223 patients were identified with TNBC or estrogen–receptor–low positive disease who received carboplatin with paclitaxel and pembrolizumab followed by ddAC per the modified KEYNOTE-522 regimen and were screened for inclusion. There were 131 patients who were excluded for not meeting inclusion criteria (Figure 1). The most common reasons for exclusion were planned schedule changes from ddAC to every three-week anthracycline and cyclophosphamide mid-treatment (*n* = 30), followed by therapy and/or surgery not being completed at time of data collection (*n* = 22). Of the 92 patients included, 51 patients (55%) received weekly carboplatin dosed with area under the curve (AUC) 1.5 with weekly paclitaxel and 41 patients (45%) received every three-week carboplatin dosed with AUC 5 and weekly paclitaxel.

Baseline demographic characteristics are summarized in Table 1. Most baseline characteristics were similar between groups. The majority of patients were 40 to 50 years of age, White, had breast cancer with ductal histology, and node-negative disease. More patients in the weekly regimen cohort had grade 3 disease compared to patients in the every three-week cohort (88.2% vs. 63.4%; *P* = 0.01), but notably less grade 2 disease (11.8% vs. 34.2%), which was a significant difference between groups (*P* = 0.01). Numerically, more patients in the weekly group had T3 sized tumors, also identified as tumors that are more than five centimeters across, (21.6% vs. 9.8%; *P* = 0.06) and stage III disease (43.1% vs. 24.4%; *P* = 0.08). Both groups had comparable or appropriate number of doses (12 vs 4 total doses of carboplatin), however patients in the every three-week cohort received a higher number of pembrolizumab doses [8-9] when compared to the weekly schedule [6-8] (*P* = 0.01). Treatment duration with each drug, measured in months, did not significantly differ between the groups.

**Table 1.** Patient demographics by treatment regimen.

Variable	Weekly regimen (*n* = 51)	Three-week regimen (*n* = 41)	Overall (*n* = 92)	*P*-value
**Age (years)**	44 [36-58]	50 [40-59]	48 [38-59]	0.13
**Female**	51 (100)	41 (100)	92 (100)	-
**Race**				
** Black**	7 (13.7)	2 (4.9)	9 (9.8)	0.12
** White**	44 (86.3)	37 (90.2)	81 (88.0)	
** More than one**	0 (0)	1 (2.4)	1 (1.1)	
** Unknown**	0 (0)	1 (2.4)	1 (1.1)	
**Ductal histology**	51 (100)	41 (100)	92 (100)	-
**Inflammatory**	5 (9.8)	2 (4.9)	7 (7.6)	0.46
**Grade**				
** I**	0 (0)	1 (2.4)	1 (1.1)	0.01
** II**	6 (11.8)	14 (34.2)	20 (21.7)	
** III**	45 (88.2)	26 (63.4)	71 (77.2)	
**Estrogen receptor status**				
** 0%**	45 (88.2)	38 (92.7)	83 (90.2)	0.73
** 1%-10%**	6 (11.8)	3 (7.3)	9 (9.8)	
**Tumor size**				
** T1**	5 (9.8)	5 (12.2)	10 (10.9)	0.06
** T2**	30 (58.8)	32 (78.1)	62 (67.4)	
** T3**	11 (21.6)	4 (9.8)	15 (16.3)	
** T4**	5 (9.8)	0 (0)	5 (5.4)	
**Nodal status**				
** Yes**	19 (37.3)	13 (31.7)	32 (34.8)	0.66
** No**	32 (62.8)	28 (68.3)	60 (65.2)	
**Clinical stage**				
** II**	29 (56.9)	31 (75.6)	60 (65.2)	0.08
** III**	22 (43.1)	10 (24.4)	32 (34.8)	

Results reported as counts (column percentages) or median [first and third quartiles].

### Outcomes

Patients in the every three-week carboplatin group were more likely to achieve a pCR at time of definitive surgery compared to those in the weekly group (70.7% vs. 47.1%, *P* = 0.003). A comparison of pCR rates during the study time frame are reported in Table 2. Across all disease stages, patients treated on a every three-week schedule experienced higher pCR rates, with rates of 68% vs. 48% in stage I (*P* = 0.19) and 80% vs. 45% in stage II (*P* = 0.12). A significant difference in pCR rates was observed among patients who were ER 0%, favoring the every three-week regimen (71% vs. 44%, *P* = 0.03). Patients who were ER 1%-10% demonstrated equivalent response rates between schedules (67% vs. 67%, *P* = 0.99). In a multivariable mixed effects model counting for repeated measures, the regimen showed a significant association with pCR rate, and the association remained significant after adjusting for age group, grade, stage, and tumor size (unadjusted: *P* = 0.02; adjusted for age, grade, tumor size, clinical stage: *P* = 0.008). (Table 3). The confounder variables were also tested as possible effect modifiers of the association between regimen and pCR; however, none of these interaction effects were statistically significant.

**Table 2.** pCR and RCB at time of surgery by treatment regimen.

Outcome	Weekly regimen (*n* = 51)	Three-week regimen (*n* = 41)	Overall (*n* = 92)	*P*-value
**Pathological CR at surgery**	24 (47.1)	29 (70.7)	53 (57.6)	0.03
**RCB at surgery (if no pCR)**				
** I**	9 (33.3)	3 (25.0)	12 (30.8)	0.74
** II**	11 (40.7)	7 (58.3)	18 (46.2)	
** III**	7 (25.9)	2 (16.7)	9 (23.1)	

Results reported as counts (column percentages). Abbreviation: AC, doxorubicin and cyclophosphamide.

**Table 3.** Multivariable logistic regression model of pCR at time of surgery.

Variable	Odds ratio of pCR (95% CI)	*P*-value
**Treatment regimen**		
** Weekly**		0.008
** Three-week**	4.59 (1.50-14.04)	
**Age (years)**		
** <50 years**		0.56
** ≥50 years**	0.75 (0.29-1.96)	
**Grade**		
** I/II**		0.007
** III**	5.76 (1.63-20.36)	
**Tumor size**		
** T1-2**		0.08
** T3-4**	0.23 (0.04-1.18)	
**Clinical stage**		
** II**		0.26
** III**	2.34 (0.54-10.13)	

OR > 1 indicates group had higher odds of pCR compared to the reference group.

Secondary outcomes were compared between treatment groups in Tables 4 and 5. For patients who did not achieve a pCR at time of surgery, the patients in each cohort predominantly had a RCB II score with 40.7% and 58.3%, followed by a RCB I score, 33.3% and 25%. Although any delay of greater than 7 days due to chemotherapy toxicities were similar between groups, numerically more patients in the weekly carboplatin group experienced treatment delays due to immunotherapy-related toxicities than every three-week carboplatin group (23.5% vs. 14.6%, *P* = 0.99). In addition to dose delays, a higher percent of patients in the weekly carboplatin group required dose reductions with carboplatin and/or paclitaxel due to chemotherapy-related toxicities (35.3% vs. 26.8%, *P* = 0.50). There was not a significant difference in grade 3 or higher neutropenia (41.2% vs. 48.8%, *P* = 0.53) nor the use of GCSF (23.5% vs. 22.0%) between the dosing schedules. More patients in the weekly group experienced iHSR. Ten patients (19.6%) in the weekly carboplatin group and 2 patients (4.9%) in the three-week carboplatin group had iHSR during their treatment (*P* = 0.06).

**Table 4.** Total number of doses and adjustments in each treatment regimen.

Outcome	Weekly regimen (*n* = 51)	Q21 day regimen (*n* = 41)	Overall (*n* = 92)	*P*-value
**Number of AC doses**	4 [4-4]	4 [4-4]	4 [4-4]	0.56
**Number of carboplatin doses**	12 [10-12]	4 [4-4]	9 [4-12]	<0.001
**Number of paclitaxel doses**	12 [12-12]	12 [12-12]	12 [12-12]	0.20
**Number of pembrolizumab doses**	8 [6-8]	8 [8-9]	8 [6.5-8]	0.01
**Delay >7d due to toxicity from carboplatin or paclitaxel**	12 (23.5)	10 (24.4)	22 (23.9)	0.99
**Delay >7d due to toxicity from pembrolizumab**	12 (23.5)	6 (14.6)	18 (19.6)	0.31
**Dose reduction of carboplatin or paclitaxel due to toxicity**	18 (35.3)	11 (26.8)	29 (31.5)	0.50

Abbreviation: AC, doxorubicin and cyclophosphamide.

**Table 5.** Toxicities by treatment regimen.

Toxicities	Weekly regimen (*n* = 51)	Q21 day regimen (*n* = 41)	Overall (*n* = 92)	*P*-value
**Any grade 3/4 neutropenia during carboplatin and paclitaxel with pembrolizumab**	21 (41.2)	20 (48.8)	41 (44.6)	0.53
**Any grade 3/4 neutropenia during AC**	14 (27.5)	8 (19.5)	22 (23.9)	0.46
**Use of GCSF**	12 (23.5)	9 (22.0)	21 (22.8)	0.99
**HSR at any time**	10 (19.6)	2 (4.9)	12 (13.0)	0.06

Results reported as counts (column percentages); Abbreviations: AC, doxorubicin and cyclophosphamide; G-CSF, growth-colony stimulating factor; HSR, hypersensitivity reaction.

## Discussion

Results of this study suggest every three-week carboplatin with weekly paclitaxel and pembrolizumab followed by ddAC and pembrolizumab in a modified KEYNOTE-522 regimen may provide higher pCR rates at the time of surgery over weekly carboplatin in patients with stage II or III TNBC. Previous studies have evaluated pCR rates following neoadjuvant treatment of early-stage TNBC but focused on the addition of pembrolizumab to standard of care chemotherapy and the sequence of AC and carboplatin with paclitaxel.^4^^,^^10^ This study is one of the first to compare the pCR rates of the two schedules of carboplatin in the KEYNOTE-522 regimen and their respective adverse effect profiles head-to-head.

Memorial Sloan Kettering Cancer Center (MSKCC) recently published a single-center, retrospective study suggesting that using ddAC in a modified KEYNOTE-522 regimen had comparable pCR rates to the KEYNOTE-522 every three-week AC dosing schedule.^10^ The KEYNOTE-522 trial design allowed treating physicians to choose if a patient received weekly or every three-week dosing of carboplatin along with weekly paclitaxel. Although neither the KEYNOTE-522 or MSKCC study compared weekly versus every three-week dosing head-to-head, they reported contrasting numerical differences in pCR rates between the groups. Numerically, KEYNOTE-522, showed greater pCR rates in the weekly carboplatin group and, conversely, MSKCC’s trial using a modified KEYNOTE-522 regimen demonstrated higher pCR rates in those who received every three-week dosing. Besides the conflicting evidence for the differences in pCR rates, these trials showed varied adverse events between the two schedules.

It is important to recognize treatment delays and dose reductions, especially for patients with TNBC, can be associated with mortality.^11^ The treatment goal should be to maintain relative dose intensity, if safe for the patient to do so, in order to improve outcomes. Although the total number of treatments were the same between our study and KEYNOTE-522, the trial did not report how many patients received dose reductions or treatment delays due to toxicities. In our study, about a quarter of patients in either group had a delay of greater than 7 days. In addition, 35% of patients in the weekly group and 26.8% in the every three-week group received a dose reduction due to carboplatin or paclitaxel toxicities. It was also found that more patients had delays of pembrolizumab caused by immunotherapy-related toxicities in the weekly group than the every three-week group. The difference between pCR rates in the studies, ours being 70.7% and KEYNOTE-522’s 64.8%, may not only have to do with a difference in cumulative carboplatin received (patients with twelve weekly doses at an AUC 1.5 receive less total milligrams than the same patient getting four every three-week doses at an AUC 5), but it could also be the difference between AC dosing schedules. Previous studies have shown that outcomes improved in patients with TNBC when providers administered ddAC when compared to traditional AC.^5^ KEYNOTE-522, however, used an every three-week dosing schedule of AC, most likely for patient convenience to receive the pembrolizumab and AC at the same time.

The secondary outcomes found in our study confirm administering carboplatin every three weeks results in comparable adverse effects to weekly administration. Patients who receive weekly carboplatin have a greater risk of iHSR due to their higher total number of doses given. Patients who receive every three-week carboplatin however tend to have higher rates of neutropenia, thrombocytopenia, neuropathy, and chemotherapy-induced nausea and vomiting (CINV).^11^ Of the patients in KEYNOTE-522, 34.6% experienced grade 3 or higher neutropenia with weekly carboplatin compared to 41.2% of patients in our weekly cohort. Although our patients had higher rates of neutropenia compared to other studies, there was no difference in the use of GCSF, therefore providing reassurance that this regimen may not be as myelosuppressive nor as financially toxic, as perceived. The consistent toxicity profile between the two carboplatin schedules should provide reassurance to providers in selecting the every-three-week dosing option for their patients.

As carboplatin has become more common in the treatment of TNBC, iHSR is a growing concern for many patients and providers. An increase in the number of total doses of carboplatin increases the risk of this toxicity, which may threaten its use in therapy altogether.^12^ Studies have shown that when patients received combined antineoplastic agents, like paclitaxel, iHSR can occur more often.^13^ Although many carboplatin reactions are mild, they can be life-threatening in some patients. In most cases, a grade 1 or grade 2 iHSR will resolve following interruption of treatment and administration of rescue medications, such as hydrocortisone, diphenhydramine, famotidine and epinephrine. Re-treatment has the potential to have serious consequences as these reactions are IgE-mediated and are likely to recur with repeated platinum exposure. Close monitoring is therefore imperative for future doses following an iHSR, and institution specific graded challenges or desensitization protocols should be utilized. Given patients receive only 4 total doses of carboplatin in the every three-week dosing versus 12 total doses in the weekly carboplatin group, the every three-week group has a significantly less risk of developing iHSR and a better chance of completing neoadjuvant therapy. As our study established that q3w carboplatin administration resulted in a greater rate of pCR with similar neutropenias, similar growth factor use, and lower rates of iHSR than weekly carboplatin, q3w carboplatin dosing should be strongly considered when for treatment of early-stage TNBC.

There are multiple limitations to this study, including that this is a retrospective study and that patients were not randomized to receive either weekly or q3w carboplatin. There may be several other factors that prescribing physicians took into account when deciding with their patients whether to pursue weekly or q3w carboplatin dosing that may not be captured with the variables described here, including patient preference, performance status, and co-morbidities. Moreover, it is difficult to collect subjective adverse effects, especially in a retrospective study. Since these toxicities are individualized to the patient, we did not collect this as we felt dose reductions better correlated with toxicity versus subjective reporting. Therefore, we did not collect data to see if patients experienced differences in severity of adverse effects between cohorts. In addition, since our institution began this regimen for the treatment of TNBC in late 2020 to 2021, it will take time to see if there are differences between overall survival and progression-free survival in this population and therefore these outcomes were not collected. A multi-institutional follow-up study needs to be conducted to see the long-term outcomes of KEYNOTE-522 and if there is a difference in the lasting impact of weekly and every three-week carboplatin dosing.

## Conclusion

In this real-world retrospective study, every three-week carboplatin compared to weekly carboplatin demonstrated greater pCR rates in a modified KEYNOTE-522 regimen of carboplatin with weekly paclitaxel and pembrolizumab followed by ddAC. There were no significant differences in adverse effect profiles, including neutropenias, between the two cohorts, except for the higher incidence of iHSR in patients who received weekly carboplatin. Further prospective, randomized trials are needed to confirm our findings on the impact of a carboplatin dosing schedule on pCR rates in patients with TNBC.

## Author contributions

Kennedi G. Satterfield, Vandergriff (Conceptualization, Data curation, Formal analysis, Investigation, Methodology, Project administration, Visualization, Writing—original draft, Writing—review & editing), Michael Berger (Conceptualization, Formal analysis, Methodology, Project administration, Supervision, Visualization, Writing—original draft, Writing—review & editing), Allison Reed (Conceptualization, Formal analysis, Methodology, Visualization, Writing—original draft, Writing—review & editing), Eric McLaughlin (Formal analysis, Methodology, Software, Writing—original draft, Writing—review & editing), Stephanie Collins (Conceptualization, Data curation, Formal analysis, Investigation, Methodology, Project administration, Supervision, Visualization, Writing—original draft, Writing—review & editing), Dionisia Quiroga (Conceptualization, Formal analysis, Methodology, Visualization, Writing—original draft, Writing—review & editing)

## Funding

This research did not receive any specific grant from funding agencies in the public, commercial, or non-for-profit sectors.

## Conflicts of interest

There are no conflicts of interest to disclose of the primary author and the results/data/figures in this manuscript have not been published elsewhere nor are they under consideration by another publisher. The following authors have declared the following disclosures.

M.B., BCOP: “Novartis advisory board within the last 6 months”.

A.R., BCOP: “Honoraria from Pharmacy Times Continuing Education (producing and editing continuing education material)”.

## Data Availability

The data underlying this article will be shared on reasonable request to the corresponding author.
